# Genetically Determined Circulating Saturated and Unsaturated Fatty Acids and the Occurrence and Exacerbation of Chronic Obstructive Pulmonary Disease—A Two-Sample Mendelian Randomization Study

**DOI:** 10.3390/nu16162691

**Published:** 2024-08-14

**Authors:** Zhao-Min Liu, Yu-Ming Chen, Chao-Gang Chen, Cheng Wang, Min-Min Li, Yu-Biao Guo

**Affiliations:** 1 Guangdong Provincial Key Laboratory of Food, Nutrition and Health, Department of Nutrition, School of Public Health, Sun Yat-sen University (North Campus), Guangzhou 510080, China; limm9@mail2.sysu.edu.cn; 2 Department of Epidemiology and Medical Statistics, School of Public Health, Sun Yat-sen University (North Campus), Guangzhou 510080, China; chenym@mail.sysu.edu.cn; 3 Department of Clinical Nutrition, Sun Yet-sen Memorial Hospital, the Second Affiliated Hospital of Sun Yat-sen University, Guangzhou 510120, Chinawangcheng@mail.sysu.edu.cn (C.W.); 4 Department of Respiratory and Critical Care Medicine, the First Affiliated Hospital of Sun Yat-sen University, Guangzhou 510080, China

**Keywords:** fatty acids, Mendelian randomization, COPD, causality

## Abstract

Research on dietary fatty acids (FAs) and lung health has reported skeptical findings. This study aims to examine the causal relationship between circulating FAs and Chronic Obstructive Pulmonary Disease (COPD) onset and exacerbation, using a two-sample Mendelian Randomization (MR) analysis. Strong and independent genetic variants of FAs were obtained from the UK Biobank of European ancestry. The exposure traits included saturated FA (SFA), poly- and mono-unsaturated FA (PUFA and MUFA), omega-3 and omega-6 PUFA, docosahexaenoic acid (DHA), and linoleic acid (LA), all expressed as total FA (TFA) percentages. Summary statistics for COPD outcomes were obtained from the FinnGen consortium including COPD, COPD hospitalization, COPD/asthma-related infections, COPD-related respiratory insufficiency, and COPD/asthma/interstitial lung disease (ILD)-related pneumonia. The inverse-variance weighted (IVW) was the primary MR approach. MR-Egger regression and MR-PRESSO were utilized to evaluate heterogeneity and pleiotropy. MR-PRESSO tests suggested no obvious horizontal pleiotropy. MR results by the IVW approach indicated that the genetically high SFA/TFA levels were associated with an increased risk of COPD/asthma/ILD-related pneumonia (OR: 1.275, 95%CI: 1.103–1.474, *p* for FDR = 0.002). No significant relationship was observed between other types of FAs and COPD outcomes. Our MR analysis suggests that there is weak evidence that the genetically predicted high SFA/TFA was associated with an increased risk of pneumonia.

## 1. Introduction

Chronic obstructive pulmonary disease (COPD) is a heterogeneous lung condition characterized by chronic respiratory symptoms with persistent and generally progressive airflow obstruction accompanied by the development of emphysema [1]. COPD patients experience a progressive decline in lung function, frequent disease exacerbations, and multiple extrapulmonary comorbidities [2]. COPD and its related complications seriously affect patients’ quality of life and bring a huge economic burden to both societies and families. Data from the Global Burden of Disease 2019, which includes 204 countries and territories, reported 212.3 million COPD patients worldwide, associated with 74.4 million disability-adjusted life years (DALYs) and 3.3 million deaths [2]. COPD has a global prevalence of 10.3% and has become the third leading cause of death (7%) worldwide [3]. Current pharmacotherapy for COPD provides symptomatic relief, but cannot halt the progressive decline in respiratory function. Nutritional interventions play a crucial role in the prevention and treatment of chronic respiratory conditions [4]. It was reported that 30–60% of COPD patients experienced malnutrition and a proper diet has become an important component of successful COPD management [4]. 

The role of fatty acids (FAs) in COPD pathogenesis has become a promising topic of research [5]. Emerging evidence increasingly highlights the preventive role of dietary unsaturated FAs in the progression of obstructive lung diseases for their anti-inflammatory properties [5]. However, observational studies and clinical trials on saturated and unsaturated FAs and lung health have reported skeptical findings [6]. Early evidence has shown that a healthy dietary pattern assessed by the Alternative Healthy Eating Index-2010 (AHEI-2010) diet score (featured as high intakes of whole grains and nuts, polyunsaturated fatty acids, while low intakes of red/processed meats and refined grains) was associated with lowered risk of COPD in both women and men [7]. In contrast, a systematic review and meta-analysis including 10 cohort, 6 case-control, and 20 cross-sectional studies, reported a null relationship between dietary intakes of omega-3 or omega-6 polyunsaturated fatty acids (PUFA) and the risk of COPD [6]. Several studies reported habitually high intakes of omega-3 and/or low intakes of omega-6 PUFA were associated with alleviated inflammation [8], improved lung function [9], and reduced respiratory symptoms [10,11] among patients of COPD or other lung disorders, while other studies did not [12,13,14]. The study among 250 clinically stable COPD patients provided the first evidence that a high dietary intake of α-Linolenic acid (ALA, one omega-3 PUFA) and a low intake of arachidonic acid (AA, one omega-6 PUFA) was associated with decreased serum inflammatory markers (i.e., TNF-α and IL-6) [8]. However, a National Health and Nutrition Examination Survey (NHANES) study among 878 COPD patients suggested no primary associations between omega-3 intake and respiratory symptoms [14]. High intakes of saturated fatty acids (SFA, typical in Western diets) could even exacerbate airway inflammation [15].

Most evidence from observational studies is open to uninvestigated or reversal confounding while clinical trials targeting COPD patients by unsaturated FA supplementation are limited. Several randomized controlled trials (RCT) [16,17] supported that the modification of dietary FA composition by increasing omega-3 but decreasing omega-6 PUFAs produced beneficial effects on improvement in lung function and decrease in bronchial inflammation, but not all [18,19], as the majority of RCTs often had a small sample size, and heterogeneously defined populations and interventions. Therefore, the impacts of FAs and COPD onset and progression remained uncertain, and a valid approach is necessitated to address the ambiguities and appreciate the causal role of FAs in the development of respiratory disorders [20]. A Mendelian randomization (MR) study is an ideal analytic approach to assess the causality of a modifiable exposure or risk factor and a clinically relevant outcome by analyzing genetic instrumental variables (IVs) with both exposure and outcome. MR analysis provides more credible evidence than conventional observational studies due to less susceptible to confounders and reverse causation as genetic variants are randomly allocated since meiosis. 

Circulating FAs can be used as objective biomarkers for assessing dietary FA consumption without self-reported biases [21]. The genetically determined circulating FAs can therefore be used to probe the lifelong impacts of various FA exposures on disease risk by MR analysis. COPD also reflects an accumulation of gene–environment interactions over the life course [22]. We thus conduct a two-sample MR analysis using summary statistics (pooled data) from the largest and latest publicly available database of Genome-wide association studies (GWAS) to investigate the causal relationship between different FAs and COPD-related outcomes. 

## 2. Materials and Methods

### 2.1. Study Design

**Figure 1:**
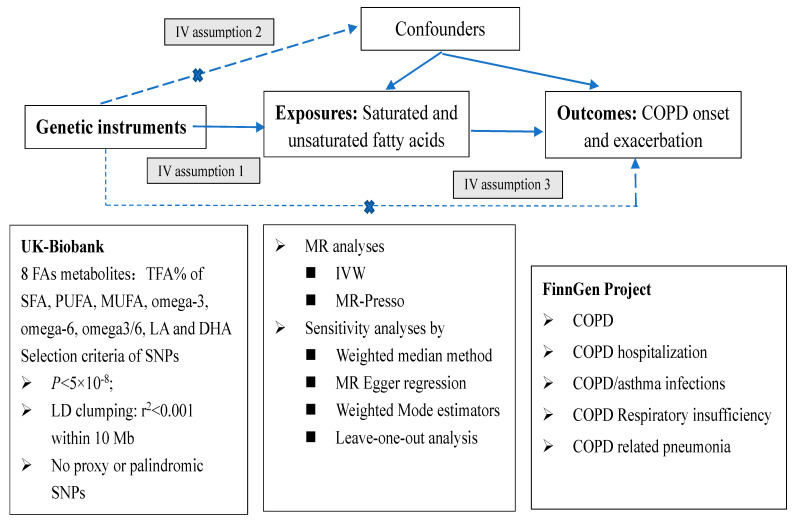
Mendelian randomization analysis on circulating fatty acids (FAs) and COPD-related outcomes. Legends: The solid arrow indicates the causal relationship in the directed acyclic graph, and the dashed line indicates the association of the estimates. Abbreviations: MR, Mendelian randomization; SNP, single-nucleotide polymorphism; TFA, total fatty acids; SFA, saturated fatty acids; PUFA, polyunsaturated fatty acids; MUFA, monounsaturated fatty acids; DHA, docosahexaenoic acid; LA, linoleic acid; IVs, instrumental variables; IVW, Inverse-variance weighted; COPD: chronic obstructive pulmonary disease; LD, linkage disequilibrium. 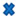 denotes the pathway is not allowed.

This was a two-sample MR analysis to explore the causal relationship between circulating saturated and unsaturated FAs with the risk of COPD onset and development (Figure 1). MR studies require the IVs to meet the following three core assumptions: (1) strong and robust correlation between genetic IVs and exposure factors (assumption 1, association hypothesis); (2) absent association between genetic IVs and confounding factors that affect the exposure and outcome relationship (assumption 2, independence hypothesis); (3) genetic variants affecting outcomes solely through exposure factors without other pathways (assumption 3, exclusivity hypothesis). The GWAS summary data for both exposures (FAs) and COPD-related phenotypes were retrieved from the IEU Open GWAS database (https://gwas.mrcieu.ac.uk/, accessed on 1 May 2024). We limited our analysis to individuals of European ancestry to minimize the potential bias from population heterogeneity. Detailed information on the GWAS identifiers is shown in Table 1. No additional ethical approval or informed consent was therefore required since all the data for the analyses were publicly available.

### 2.2. Data Sources for Exposure Factors and Outcomes

Previous research has categorized FAs into four primary types: total FAs (TFAs), saturated FAs (SFAs), poly- and monounsaturated FAs (PUFAs and MUFAs). This study further analyzed two specific PUFAs, Omega-3 and Omega-6 PUFAs, which are predominantly comprised of docosahexaenoic acid (DHA) and linoleic acid (LA), respectively. The exposure traits of FAs in our analysis were all adjusted by TFA (TFA percentages), which included SFA/TFA, PUFA/TFA, MUFA/TFA, omega-3/TFA, omega-6/TFA, omega-6/omega-3 FAs, LA/TFA, and DHA/TFA. Summary data on these eight circulating FAs (exposure factors) were obtained from the largest available GWAS, the UK Biobank (UKB). UKB is a renowned population-based cohort consisting of half a million British participants aged 40 to 69 years and recruited from 2006 to 2010 [23]. All the participants completed questionnaires and health assessments, and provided samples for metabolites testing and genotyping. Metabolites of FAs were quantified using high-throughput nuclear magnetic resonance (NMR) spectroscopy by metabolomic analysis, which was available for approximately one-third of participants sampled randomly. Almost all the samples (97%) pertain to the recruitment visit [24]. The analyses included 115,006 European descent of participants and 11,590,399 quality-controlled genetic variants [24]. These genetic associations had already been adjusted for population structure, fasting time, and gender. 

The outcomes of interest were COPD initiation and progression, which included COPD, COPD hospital admissions, COPD/asthma-related infections, COPD-related respiratory insufficiency, and COPD/asthma/interstitial lung disease (ILD)-related pneumonia or pneumonia-derived septicemia, which was defined in FinnGen dataset as an acute, acute and chronic, or chronic inflammation focally or diffusely affecting the lung parenchyma, caused by an infection in one or both of the lungs by bacteria, viruses, fungi, or mycoplasma. Among them, COPD-related hospitalization, respiratory insufficiency, and infections were implications of COPD exacerbations. The GWAS data for COPD outcomes were procured from the most recently published database of the FinnGen consortium R9 (https://r9.finngen.fi/, accessed on 1 May 2023), encompassing 18,266 cases and 311,286 controls. FinnGen was a research project in genomics and personalized medicine launched in Finland in 2017. The project combined genotype data from Finnish biobanks and digital health record data from Finnish health registries from 500,000 Finnish participants. Its database consists of a rich compilation of prospective epidemiological cohorts, disease-based cohorts, and hospital biobank samples. Summary data for COPD outcomes have been adjusted for age, gender, smoking, genotyping array, and 10 principal components. There was no population overlap between the above two databases.

### 2.3. Selection of Instrumental Variables

We used standard criteria for IV selection with genome-wide significance of *p* < 5 × 10^−8^. All the IVs were clumped to avoid linkage disequilibrium (LD) and ensure independence of IVs with r^2^ < 0.001 and genetic distance >10 MB. Proxy single-nucleotide polymorphisms (SNPs) were not applied, and palindromic SNPs were excluded. The strength of the IVs was assessed by F-statistics, calculated as (beta/se)^2^ of exposure IVs [25]. F-statistic values above 10 suggest weak IV bias being unlikely. We then harmonized the exposure and outcome to investigate the impacts of genetic IVs of FAs on COPD outcomes after the exclusion of palindromic and incompatible SNPs. 

### 2.4. Statistical Analysis by Mendelian Randomization

Data processing and analysis were performed using R version 4.3.0, along with Storm Statistical Platform (www.medsta.cn/software, accessed on 1 August 2024). The R packages of ‘devtools’, ‘TwoSampleMR’ (0.5.8), and ‘MRPRESSO’ were applied for data analysis. This study conducted analyses for 8 subtypes of FAs and 5 COPD-related outcomes. False discovery rate (FDR) by Benjamini–Hochberg method was applied to correct *p* values of MR estimates if they were less than 0.1.

In this two-sample MR analysis, inverse-variance weighting (IVW) was the primary method to estimate the impact of genetically predicted FAs on COPD outcomes. Additional statistical techniques such as MR-Egger regression, Weighted Median (WM), Simple Mode, and Weighted Mode estimators were supplemented to verify the robustness of the main results. In addition to an estimate of causal effect, MR-Egger was utilized to test for directional pleiotropy with a non-zero intercept suggesting horizontal pleiotropy. Cochran Q statistic by IVW approach was applied to assess the heterogeneity of the contained IVs. In cases where heterogeneity was present, causality was determined using a random effect model. The MR pleiotropic residual sum and outlier test (MR-PRESSO) was employed to test the horizontal pleiotropy [26], and suspected IVs of significant pleiotropy were removed and causality was then reassessed. Sensitivity analysis by leave-one-out analysis was performed by deleting one SNP at a time to examine the influence of a single SNP and demonstrate the robustness of the MR estimates. Funnel plots were illustrated to identify outliers and the asymmetry suggested heterogeneity of IVs.

We performed a replication MR analysis using absolute FAs (without TFA adjustment) GWAS data as the exposure traits, which were also obtained from the IEU database (UK-Biobank, year 2020, 114,999 European participants and 12,321,875 SNPs) to cross-validate the reliability of our results [27]. The GWAS-ID included met-d-SFA, met-d-MUFA, met-d-PUFA, met-d-Omega_3, met-d-Omega_6, and met-d-TFA. Additional sensitivity analyses were conducted to explore the impacts of unsaturated traits of FAs (met-c-847, Average number of methylene groups per double bond; met-c-844, Ratio of bisallylic groups to double bonds) on the COPD outcomes. 

## 3. Results

The genetic information (such as GWAS-ID, chrome position, phenotype, effect allele, beta (effect size) standard error (SE), F statistics, etc.) for selected SNPs after harmonization of exposure and outcome IVs was presented in Supplemental Tables S1–S8. All the F values of SNPs were above 30, suggesting the influence from weak IVs unlikely. Cochran’s Q test by IVW approach suggested it was mostly homogenous, and heterogeneity was only found in several IVs: DHA/TFA with COPD (*p* = 0.012); LA/TFA and DHA/TFA with COPD hospitalization (*p* = 0.035 and 0.033, respectively); and SFA/TFA and DHA/TFA with COPD/asthma-related infections (*p* = 0.010 and 0.004, respectively) (Table 2). IVW random-effect (RE) models were therefore applied for these instrumental heterogeneities. MR-PRESSO tests (Table 2) did not find significant horizontal pleiotropy in all the analyses (all *p* > 0.05), although MR-Egger Intercept suggested a potentially directional pleiotropy in the associations of LA/TFA with COPD (*p* = 0.032) and COPD-related hospitalization (*p* = 0.028). 

A significant causal relationship was only found between SFA/TFA and COPD/asthma/ILD-related pneumonia even after FDR adjustment. MR analysis by the IVW approach showed the genetically high SFA/TFA was associated with an increased risk of pneumonia (OR = 1.275, 95%CI: 1.103–1.474, *p* for FDR = 0.002). The genetically determined omega-6/TFA had a marginally reduced risk of pneumonia (OR = 0.908, 95%CI: 0.832–0.990, *p* for FDR = 0.055 by IVW approach). No significant associations were observed between other FAs and COPD outcomes, although marginal significance was observed between SFA/TFA and COPD, and omega-6/TFA and COPD/asthma-related infections before FDR adjustment. 

### Visualized Results and Sensitivity Analyses

The results of MR analysis on the associations of circulating FAs with COPD outcomes are visualized in Supplemental Figures S1–S4. Scatter plots (Supplemental Figure S1) illustrated the effect size for various MR approaches. Forest plots (Supplemental Figure S2) displayed the estimated size of individual SNP impacts. The leave-one-out plot (Supplemental Figure S3) indicated the effects of individual IVs of FAs on COPD variables. The IVW estimates were slightly modified upon removal of rs174564 but without significantly skewed overall findings. The funnel plots (Supplemental Figure S4) verified the balanced distribution of individual SNP effects. 

Sensitivity analyses applying absolute FAs as exposure traits showed similar results with those of percentage data (all *p* > 0.05). The genetically predicted absolute SFA levels were marginally associated with an increased risk of pneumonia with an OR of 1.687 (95%CI: 0.946–3.007, *p* = 0.076). The MR results of the unsaturated traits of FAs (met-c-847 and met-c-844) with COPD outcomes showed overall non-significant findings (all *p* > 0.05). 

## 4. Discussion

### 4.1. Summary of Current Findings and Implications

The study aimed to directly evaluate the causal associations of saturated and unsaturated fatty acids with COPD risk and aggregation from the perspective of genetic epidemiology. We only found weak evidence (*p* = 0.002) that the genetically predicted high SFA/TFA was associated with an increased risk of pneumonia. The results remained generally non-significant when using genetically predicated absolute levels of FAs, or unsaturated traits as exposures. Up to now, two MR studies have testified the causality of circulating FAs with lung health, and both reported mostly negative associations. One MR study [28] reported a null association of various saturated and unsaturated FAs with the risk of asthma, but three genetically determined unsaturated traits (the average number of methylene groups, the ratio of bis-allylic groups to double bonds, and the ratio of bis-allylic groups to TFA) were causally associated with the decreased risk of asthma. The other MR findings [9], though, reported a protective effect of high circulating omega-3 FA, mostly DHA, on lung function, but no significant relationship was found for airway obstruction. 

To our knowledge, this is the first MR study exploring the relationship of saturated and unsaturated circulating FAs with COPD-related outcomes, not only the disease risk but also the progression. The associations between unsaturated FAs and COPD outcomes have been widely studied in observational studies and clinical trials; however, no consensus has yet been reached. Our two-sample MR analysis utilized the largest and most updated publicly available GWAS summary data from the UK-Biobank and FinnGen, and found little evidence that the genetically predicted unsaturated FAs had significant associations with COPD-related outcomes. The weak causality between SFA/TFA with COPD-related outcomes should be confirmed in large-scale prospective studies.

### 4.2. Results Explanation of SFA and Omega-6 FA with COPD Outcomes

The relationship between SFA and lung health remains uncertain in observational studies [29,30,31,32]. Several studies have reported an inverse relationship between dietary or circulating SFA with lung function (FEV_1_/FVC ratio) [29,32] while others have not [30,31]. In vitro studies have suggested that SFA could trigger a pro-inflammatory response to infection via p38 MAPK signaling, which would lead to more severe airway inflammation and increase the severity of asthma exacerbations [33]. These findings are supported by our MR results that high circulating SFA/TFA might increase the risk of pneumonia. Observational studies have implicated that the chain length and food source of SFA [30,34] could affect the effects of SFA on lung health as long-chain SFAs showed more profound pro-inflammatory properties [35] and SFA from dairy products could alleviate CT-defined emphysema [34].

Previous MR analysis showed the traits related to the degree of unsaturation of FAs played a protective role in the risk of asthma [28]. The genetically instrumented higher average number of methylene groups or the lower ratio of bis-allylic groups to double bonds or TFA were causally associated with a lowered risk of asthma [28]. However, our sensitivity analyses found the IVs of methylene groups, double bonds, and bis-allylic groups were unlikely to affect COPD initiation and progression. The relatively weak causality of our findings on SFA/TFA and pneumonia might be due to the heterogeneous outcome variable of COPD/asthma/ILD-related pneumonia. More precise COPD phenotypes or comorbidities diagnosis are warranted to confirm the findings. In addition, some COPD traits, such as COPD-related respiratory insufficiency and COPD hospital admissions in our MR analysis had a small number of cases, which could result in false negative errors. The insufficient statistical power of using a dichotomous (incidence of COPD outcomes) instead of a continuous outcome [9] (lung function) might also contribute to the mostly non-significant findings. In addition, the poor appetite in patients with aggregated COPD had insufficient consumption of dietary unsaturated FAs but an increased pro-inflammatory status. 

Our MR results suggest a marginally inverse association between the genetically determined omega-6/TFA and the risk of pneumonia. Although there is evidence that a high omega-6 FA diet inhibits the anti-inflammatory and inflammation-resolving effect of the omega-3 FA [36], increasing evidence [37,38] has reported the beneficial effects of omega-6 PUFA on a range of chronic disorders (cardiovascular disease, diabetes, chronic renal disease, and even lung cancer) without a negative inflammation response. Our findings implicated that the high omega-6 PUFA to TFA ratio might be favorable for lowering the risk of pneumonia. Long-chain PUFAs have been approved for playing a role in immune development by the synthesis of different mediators including prostaglandins, leukotrienes, thromboxanes, protectins, and resolvins, which can interfere with viruses and modulate inflammation, which is of clinical significance in preventing respiratory tract infections and allergic sensitization [39,40]. In addition, COPD/asthma/pneumonia patients might have an increased energy expenditure and demand due to the increased work of breathing. The worsening lung disorder might be alleviated when unsaturated FAs are used as the energy substrate.

Early MR studies reported that Fatty Acid Desaturase 1 (FADS1) was one of the most pleiotropic loci related to long-chain unsaturated FAs [28,41]. Our findings by leave-one-out analysis also suggested that removal of the rs174564 gene might lead to results’ fluctuation but the overall findings remained non-significant. The rs174564 gene is a prime variant in the 3′UTR region of FADS1 and plays a significant role in omega-3 FA production [42]. Previous MR studies have reported conflicting findings regarding the relationship between circulating PUFA and health outcomes, which are seemingly attributable to the inclusion of genetic variant mapping to the FADS locus in the analyses [9,28]. In-depth and rigorous clinical research based on the FADS’s genotype is needed to better understand the underlying mechanisms.

## 5. Strengths and Limitations

The notable strength of our study was the utilization of summary data from two of the largest European biobanks, which have a considerable sample size and no population overlap, to explore the causal relationship between circulating FAs and COPD risks and progress. Additionally, individual subtypes of FAs were reported as percentages of TFA rather than absolute concentrations, which could reduce the bias due to measurement errors [43]. We conducted a range of sensitivity analyses to unearth potential causal connections and improved the study’s credibility and reliability. The findings remained robust across multiple analytical approaches and exhibited no significant horizontal pleiotropy by the MR-PRESSO approach. 

Some limitations should be noted. First, while MR-PRESSO failed to find evidence of pleiotropy, it was still possible that pleiotropy might be present and potentially bias the results. In addition, some outcome traits might be heterogenous including COPD, asthma, pneumonia, or other pulmonary disorders. Therefore, the causal relationship inferred between FAs and COPD needs to be revisited. Second, the analyses were relied on publicly pooled data from GWAS and lacked individual-level data. The potentially non-linear relationships between exposure and outcome, and subgroup analyses by age, gender, and comorbidities could not be found accordingly. Third, survivor bias could not be precluded as elderly patients might be more susceptible to COPD adversaries than those who were younger. In addition, MR is a causality test rather than determining underlying mechanisms and exact clinical significance. The findings need to be confirmed through RCTs with rigorous control, and potential mechanisms should be verified by experimental evidence. In addition, peripheral blood was chosen as the sample source for the metabolomics GWAS in UK-biobank. However, it is unclear whether FA concentrations have similar roles in bronchoalveolar lavage fluid, because of the reported enrichment of FAs in airway epithelial cells. Finally, the GWAS data used in our study were from cohorts of European ancestry, and the findings might not be directly extrapolated to non-European ancestry as prospective studies have reported obvious ethnical differences in PUFA consumption and health outcomes [44]. Black and Asian populations might obtain more benefits from fish consumption or omega-3 supplementation to reduce cardiac events or cognitive decline than other ethnicities [44]. 

## 6. Conclusions

Our findings by the two-sample MR approach showed weak evidence that the genetically predicted high SFA/TFA was associated with an increased risk of pneumonia. A larger sample size of COPD cases and more precise COPD phenotypes are necessary to re-evaluate the causal relationship of circulating FAs on COPD occurrence and development. Future studies with the utilization of machine learning approach and comprehensive bioinformatics are promising to accurately predict COPD risk or prognosis.

## Figures and Tables

**Table 1:** GWAS information of various fatty acids and COPD outcomes for Mendelian randomization analyses.

GWAS ID	Trait	Year	Population	Sample Size (Case/Control)	Number of SNPs	PMID/Consortium
**Fatty acids**						
ebi-a-GCST90092981	SFA/TFA	2022	European	115,006	11,590,399	35213538/UKbiobank
ebi-a-GCST90092929	MUFA/TFA	2022	European	115,006	11,590,399	35213538/UKbiobank
ebi-a-GCST90092941	PUFA/TFA	2022	European	115,006	11,590,399	35213538/UKbiobank
ebi-a-GCST90092932	Omega-3/TFA	2022	European	115,006	11,590,399	35213538/UKbiobank
ebi-a-GCST90092935	Omega-6/TFA	2022	European	115,006	11,590,399	35213538/UKbiobank
ebi-a-GCST90092934	Omega-6/omega-3	2022	European	115,006	11,590,399	35213538/UKbiobank
ebi-a-GCST90092881	LA/TFA	2022	European	115,006	11,590,399	35213538/UKbiobank
met-d-DHA_pct	DHA/TFA	2020	European	114,999	12,321,875	UKbiobank
**COPD-related outcomes**						
finn-b-J10_COPD	COPD	2021	European	(6915/186,723)	16,380,382	FinnGen
finn-b-COPD_HOSPITAL	COPD, hospital admissions	2021	European	(6500/212,292)	16,380,466	FinnGen
finn-b-COPD_ASTHMA_INFECTIONS	COPD/asthma-related infections	2021	European	(58,925/159,867)	16,380,466	FinnGen
finn-b-COPD_INSUFFICIENCY	COPD-related respiratory insufficiency	2021	European	(1031/186,723)	16,380,365	FinnGen
finn-b-PULM_PNEUMONIA_SEPSIS	COPD/asthma/ILD-related pneumonia or pneumonia-derived septicemia	2021	European	(27,715/159,867)	16,380,352	FinnGen

GWAS, Genome-Wide Association Study; PMID: PubMed Unique Identifier; SNP, single-nucleotide polymorphism; TFA, total fatty acids; SFA, saturated fatty acids; MUFA, monounsaturated fatty acids; PUFA, polyunsaturated fatty acids; DHA, docosahexaenoic acid; LA, linoleic acid; COPD, chronic obstructive pulmonary disease; ILD, interstitial lung disease.

**Table 2:** Mendelian randomization estimates of various fatty acids and COPD outcomes using publicly available data.

Outcome/Exposure	No. SNPs	MR-PRESSO	MR-Egger Pleiotropy	Heterogeneity (IVW)		IVW		WM (Weighted Median)	
COPD/FAs		*p*	Intercept (*p*)	Q-Value (*p*)		OR (95%CI)	*p* (FDR)	OR (95%CI)	*p* (FDR)
**COPD**									
SFA/TFA	21	0.237	0.021 (0.310)	24.74 (0.211)	FE	1.254 (0.960–1.637)	0.097 (0.162)	1.475 (1.059–2.054)	0.022 (0.044)
MUFA/TFA	48	0.069	0.0016 (0.808)	64.90 (0.043)	RE	1.015 (0.884–1.165),	0.832	0.930 (0.774–1.117)	0.437
PUFA/TFA	38	>0.05 (NA)	−0.005 (0.574)	56.35 (0.022)	RE	0.944 (0.781–1.141)	0.552	0.962 (0.752–1.232)	0.761
omega-3/TFA	28	0.928	−0.006 (0.319)	18.85 (0.875)	FE	1.044 (0.962–1.134)	0.301	1.051 (0.958–1.153)	0.293
omega-6/TFA	39	0.173	−0.002 (0.789)	45.44 (0.190)	FE	0.948 (0.794–1.113)	0.558	0.854 (0.661–1.104)	0.228
omega-6/omega-3	24	0.837	0.003 (0.628)	18.01 (0.757)	FE	0.956 (0.879–1.040)	0.299	0.950 (0.862–1.047)	0.301
LA/TFA	28	0.107	−0.0167 (0.032)	39.55 (0.05)	RE	0.900 (0.752–1.077)	0.250	0.911 (0.755–1.099)	0.330
DHA/TFA	21	0.164	−0.016 (0.112)	37.05 (**0.012**)	RE	0.999 (0.850–1.174)	0.991	1.059 (0.929–1.207)	0.390
**COPD, hospital admissions**									
SFA/TFA	21	0.195	0.024 (0.265)	25.68 (0.177)	RE	1.227 (0.929–1.622)	0.149	1.294 (0.908–1.845)	0.154
MUFA/TFA	49	0.115	0.003 (0.694)	60.46 (0.107)	FE	1.029 (0.900–1.176)	0.678	0.949 (0.792–1.137)	0.571
PUFA/TFA	39	0.065	−0.006 (0.494)	52.06 (0.064)	FE	0.938 (0.781–1.127)	0.494	0.982 (0.774–1.245)	0.880
omega-3/TFA	28	0.937	−0.006 (0.266)	18.25 (0.895)	FE	1.030 (0.947–1.120)	0.493	1.037 (0.943–1.142)	0.453
omega-6/TFA	40	0.169	−0.005 (0.510)	47.39 (0.168)	FE	0.932 (0.777–1.118)	0.450	0.905 (0.689–1.187)	0.470
omega-6/omega-3	24	0.780	0.004 (0.571)	19.62 (0.664)	FE	0.972 (0.892–1.060)	0.524	0.962 (0.874–1.059)	0.433
LA/TFA	28	0.051	−0.018 (**0.028**)	41.69 (**0.035**)	RE	0.911 (0.754–1.100)	0.332	0.920 (0.756–1.119)	0.406
DHA/TFA	20	0.937	−0.011 (0.567)	31.79 (0.033)	RE	0.778 (0. 556–1.088)	0.143	0.850 (0.570–1.267)	0.426
**COPD/asthma-related infections**									
SFA/TFA	21	NA	0.00027 (0.978)	37.71 (0.010)	RE	1.087 (0.955–1.236)	0.206	1.042 (0.902–1.205)	0.575
MUFA/TFA	49	0.061	0.001 (0.640)	65.06 (0.051)	FE	1.030 (0.976–1.086)	0.282	1.005 (0.937–1.077)	0.895
PUFA/TFA	39	0.193	0.003 (0.251)	44.79 (0.208)	FE	0.978 (0.916–1.043)	0.496	0.992 (0.902–1.091)	0.873
omega-3/TFA	28	0.294	−0.0002 (0.930)	34.72 (0.146)	FE	1.011 (0.975–1.048)	0.563	1.007 (0.970–1.045)	0.714
omega-6/TFA	40	0.261	0.0001 (0.970)	43.49 (0.286)	FE	0.940 (0.879–1.004)	0.065 (0.113)	0.892 (0.806–0.987)	0.027 (0.051)
omega-6/omega-3	24	0.168	−0.0005 (0.870)	33.71 (0.069)	FE	0.991 (0.952–1.031)	0.651	0.993 (0.958–1.029)	0.693
LA/TFA	28	0.269	0.0005 (0.852)	32.85 (0.202)	FE	0.981 (0.920–1.046)	0.549	0.985 (0.915–1.061)	0.690
DHA/TFA	21	0.065	−0.001 (0.775)	41.05 (0.004)	RE	0.999 (0.935–1.068)	0.976	1.009 (0.959–1.061)	0.729
**COPD-related respiratory insufficiency**									
SFA/TFA	21	0.234	0.028 (0.572)	24.54 (0.220)	FE	1.291 (0.677–2.459)	0.438	1.379 (0.621–3.064)	0.742
MUFA/TFA	49	0.259	−0.006 (0.677)	53.96 (0.257)	FE	1.157 (0.858–1.560)	0.339	0.968 (0.642–1.459)	0.877
PUFA/TFA	39	0.118	−0.0002 (0.991)	48.57 (0.117)	FE	0.919 (0.604–1.397)	0.692	1.032 (0.574–1.855)	0.917
omega-3/TFA	28	0.951	−0.013 (0.352)	17.44 (0.920)	FE	1.031 (0.844–1.258)	0.766	1.054 (0.844–1.315)	0.644
omega-6/TFA	40	0.328	0.007 (0.700)	43.08 (0.301)	FE	0.805 (0.535–1.213)	0.300	0.708 (0.393–1.278)	0.252
omega-6/omega-3	24	0.965	0.002 (0.904)	13.23 (0.947)	FE	0.943 (0.747–1.190)	0.547	0.943 (0.747–1.190)	0.620
LA/TFA	28	0.157	−0.025 (0.180)	36.78 (0.099)	FE	0.798 (0.524–1.215)	0.293	0.922 (0.591–1.440)	0.722
DHA/TFA	21	0.618	−0.025 (0.178)	14.68 (0.794)	FE	0.952 (0.714–1.269)	0.738	1.062 (0.776–1.454)	0.708
**COPD/asthma/ILD-related pneumonia**									
SFA/TFA	21	0.158	−0.007 (0.503)	26.64 (0.146)	FE	1.275 (1.103–1.474)	0.001 (0.002)	1.186 (0.987–1.425)	0.069 (0.120)
MUFA/TFA	49	0.505	0.0013 (0.646)	45.99 (0.555)	FE	0.984 (0.926–1.046)	0.607	0.942 (0.858–1.035)	0.212
PUFA/TFA	39	0.328	0.0007 (0.843)	39.47 (0.404)	FE	1.002 (0.924–1.087)	0.965	1.088 (0.960–1.232)	0.186
omega-3/TFA	28	0.617	0.001 (0.636)	25.30 (0.557)	FE	1.041 (0.998–1.087)	0.065	1.034 (0.986–1.085)	0.164
omega-6/TFA	40	0.300	−0.0002 (0.958)	42.00 (0.342)	FE	0.908 (0.832–0.990)	0.029 (0.055)	0.873 (0.765–0.996)	0.043 (0.078)
omega-6/omega-3	24	0.324	0.0016 (0.678)	28.55 (0.196)	FE	0.967 (0.921–1.015)	0.177	0.966 (0.919–1.017)	0.187
LA/TFA	28	0.100	−0.002 (0.606)	38.73 (0.067)	FE	0.928 (0.846–1.018)	0.114	0.937 (0.849–1.034)	0.193
DHA/TFA	21	0.554	0.001 (0.796)	21.88 (0.347)	FE	1.048 (0.983–1.118)	0.153	1.051 (0.984–1.122)	0.141

SFA: saturated fatty acids; TFA: total fatty acids; MUFA, monounsaturated fatty acids; PUFA, polyunsaturated fatty acids; LA: linoleic acid; DHA, Docosahexaenoic acid; FE: fixed effects; RE: random effects; COPD: chronic obstructive pulmonary diseases; OR, odds ratio; SNP, single-nucleotide polymorphism; IVW, inverse variance weighted; GWAS, genome-wide association studies; ILD, interstitial lung disease; NA, not applicable. FDR: false discovery rate. FDR by B-H method was applied to correct *p* values if *p* < 0.1 and reported as *p* (FDR).

## Data Availability

The names of the GWAS datasets can be found in Table 1 and the Methods Section of the article. The GWAS summary statistics of UK-Biobank and FinnGen datasets are available on the IEU OpenGWAS database (https://gwas.mrcieu.ac.uk/, accessed on 1 May 2024). The associated SNPs can be accessed in the GWAS Catalog (https://www.ebi.ac.uk/gwas/home, accessed on 1 August 2024).

## Supplementary Materials

The following supporting information can be downloaded at: https://www.mdpi.com/article/10.3390/nu16162691/s1. Table S1: SNPs on SFA and COPD; Table S2: SNPs on MUFA and COPD; Table S3: SNPs on PUFA and COPD; Table S4: SNPs on Omega-3 PUFA and COPD; Table S5: SNPs on Omega-6 PUFA and COPD; Table S6: SNPs on Omega-3/omega-6 PUFA and COPD; Table S7: SNPs on LA and COPD; Table S8: SNPs on DHA and COPD; Figure S1: Scatter plots of various fatty acids and COPD related outcomes; Figure S2: Forrest plots on various fatty acids and COPD related outcomes; Figure S3: Leave-one-out plots on various fatty acids and COPD related outcomes; Figure S4: Funnel plots on various fatty acids and COPD related outcomes. For Supplemental Figures S1–S4, each figure has below 8 parts from (a) to (h) which are combined as a single composite file. (a) SFA/TFA and COPD, COPD hospitalization, COPD infections, COPD insufficiency and pulmonary infections, (b) MUFA/TFA and COPD, COPD hospitalization, COPD infections, COPD insufficiency and pulmonary infections, (c) PUFA/TFA and COPD, COPD hospitalization, COPD/asthma related infections, COPD insufficiency and pulmonary infections, (d) Omega-3/TFA and COPD, COPD hospitalization, COPD infections, COPD insufficiency and pulmonary infections, (e) Omega-6/TFA and COPD, COPD hospitalization, COPD infections, COPD insufficiency and pulmonary infections, (f) Omega-6/omega-3 ratio and COPD, COPD hospitalization, COPD/asthma related infections, COPD insufficiency and pulmonary infections, (g) DHA and COPD, COPD hospitalization, COPD/asthma related infections, COPD insufficiency and pulmonary infections, (h) LA and COPD, COPD hospitalization, COPD/asthma related infections, COPD insufficiency and pulmonary infections.
