# Supplementary material for: Genetically Determined Circulating Saturated and Unsaturated Fatty Acids and the Occurrence and Exacerbation of Chronic Obstructive Pulmonary Disease—A Two-Sample Mendelian Randomization Study
Source: Nutrients. 2024 Aug 14;16(16):2691. doi: 10.3390/nu16162691 (PMC11356979; doi:10.3390/nu16162691)
Supplement: Supplementary file 1 [file nutrients-16-02691-s001.zip › Supplemental Figure 1-4 of FA and COPD 202408.pdf]

Supplemental Figures

Supplemental Figure S1. Scatter plots of various fatty acids and COPD related outcomes

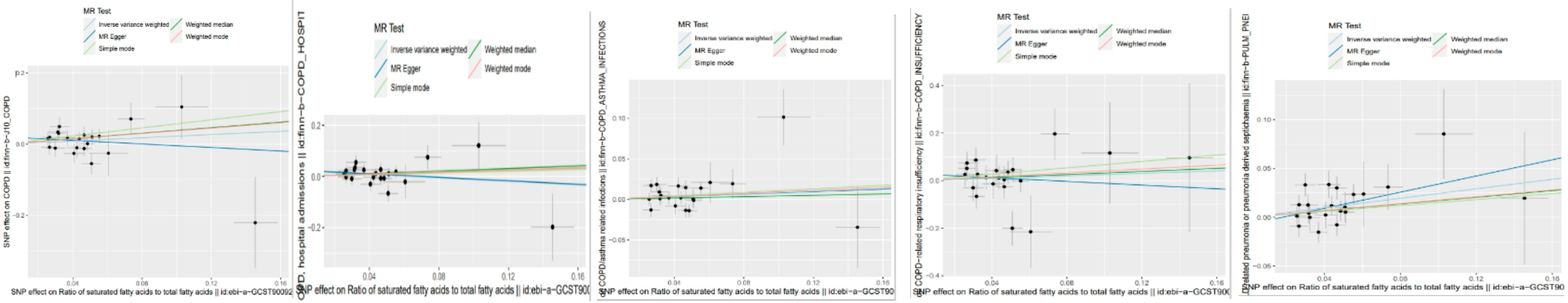

a) SFA/TFA and COPD, COPD hospitalization, COPD infections, COPD insufficiency and pulmonary infections

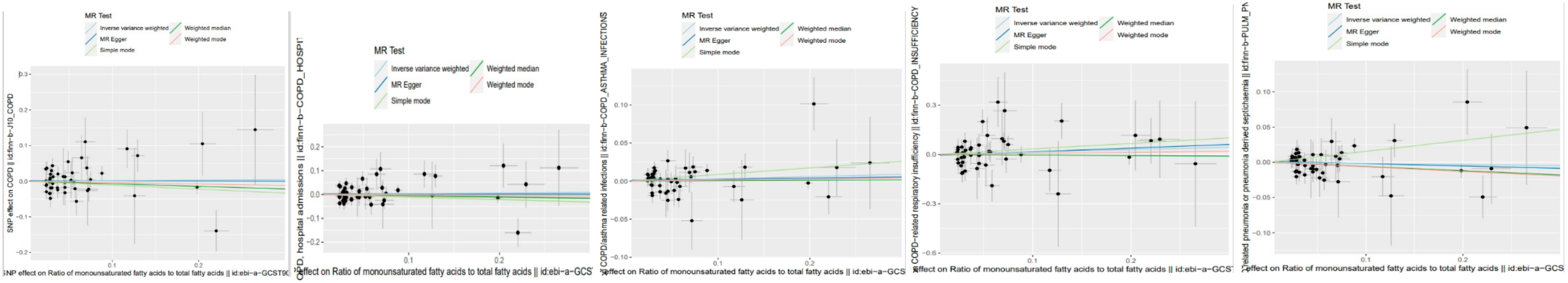

b) MUFA/TFA and COPD, COPD hospitalization, COPD infections, COPD insufficiency and pulmonary infections

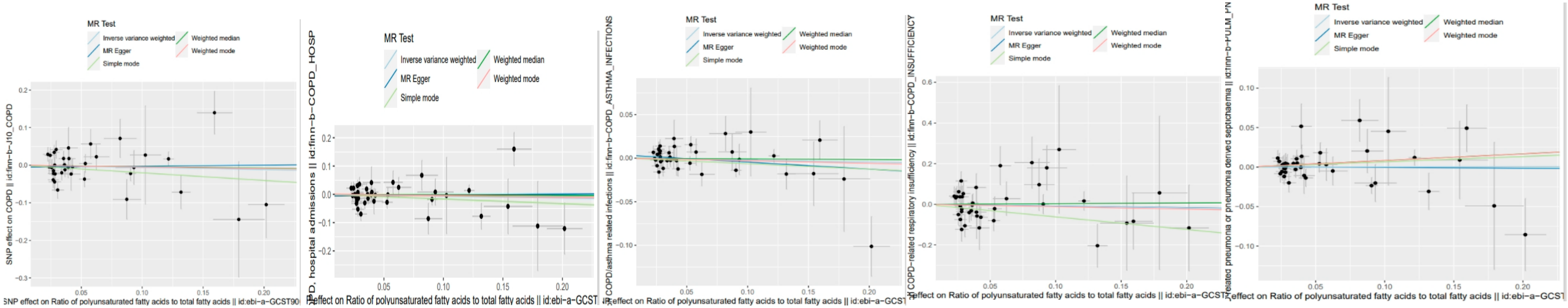

c) PUFA/TFA and COPD, COPD hospitalization, COPD/asthma related infections, COPD insufficiency and pulmonary infections

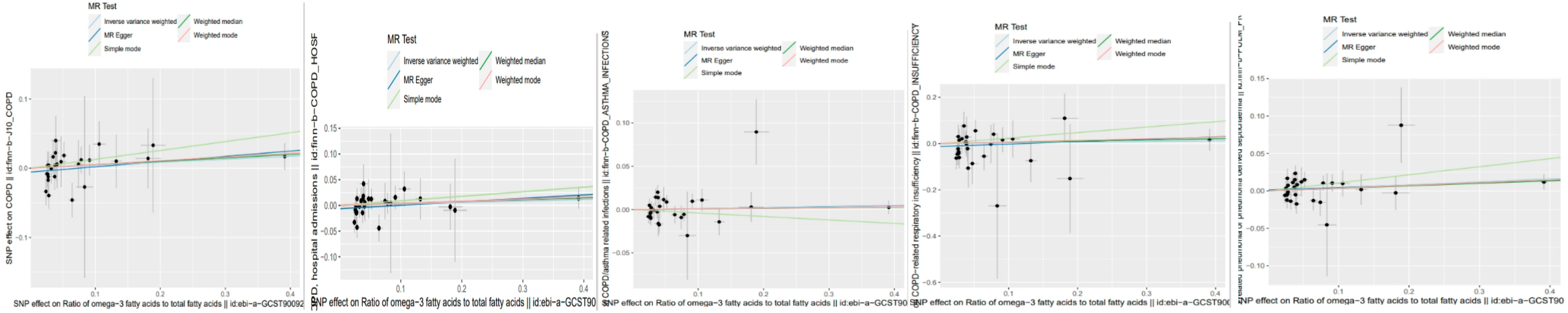

d) Omega-3/TFA and COPD, COPD hospitalization, COPD infections, COPD insufficiency and pulmonary infections

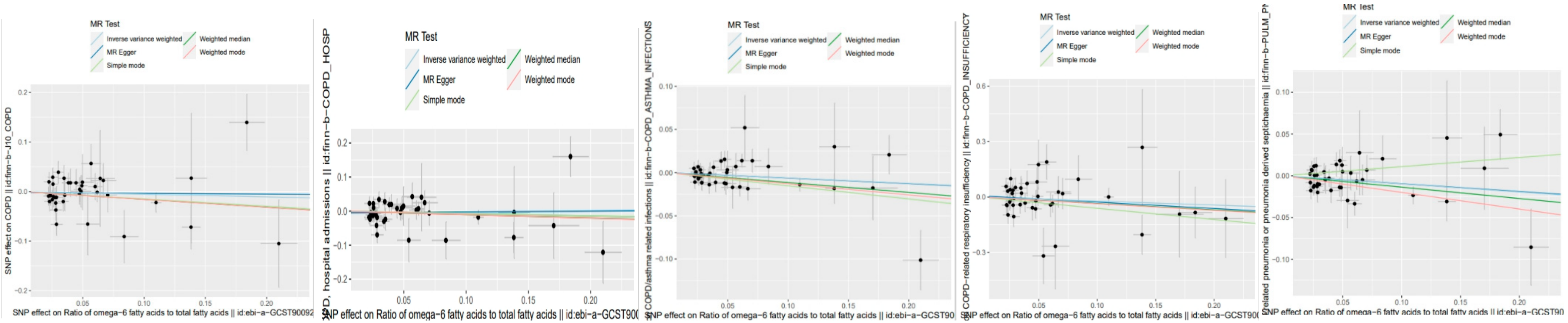

e) Omega-6/TFA and COPD, COPD hospitalization, COPD infections, COPD insufficiency and pulmonary infections

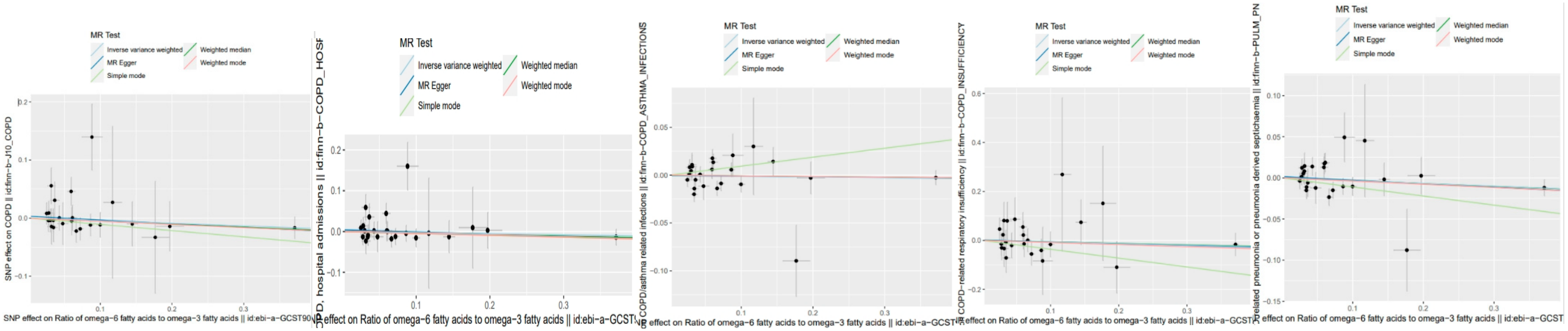

f) Omega-6/omega-3 ratio and COPD, COPD hospitalization, COPD/asthma related infections, COPD insufficiency and pulmonary infections

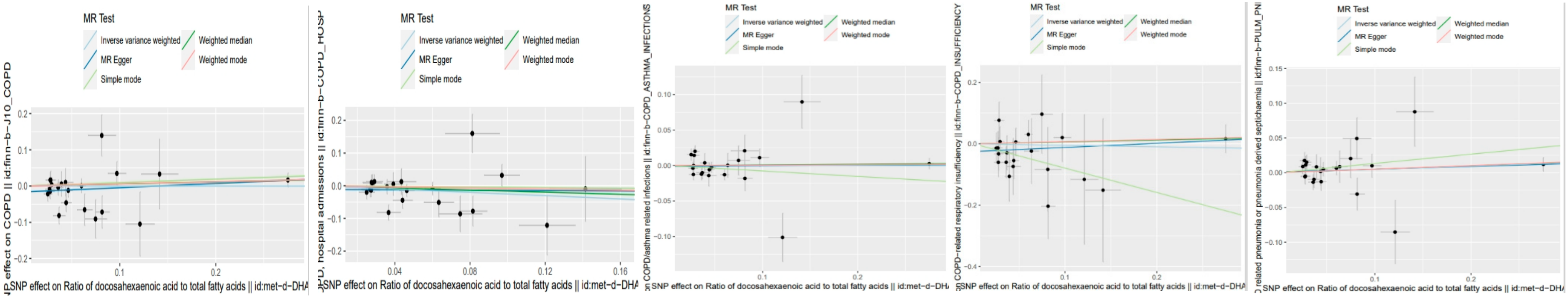

g) DHA and COPD, COPD hospitalization, COPD/asthm a related infections, COPD insufficiency and pulmonary infections

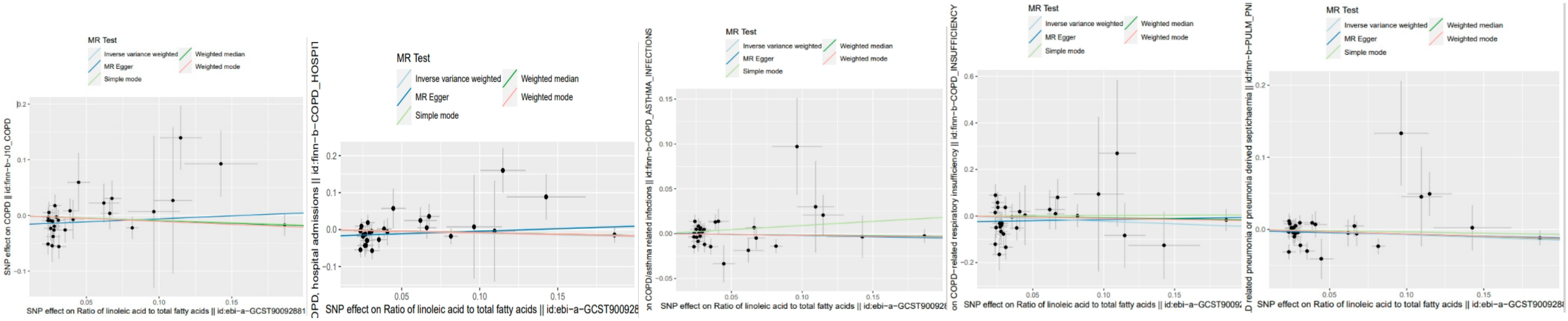

h) LA and COPD, COPD hospitalization, COPD/asthma related infections, COPD insufficiency and pulmonary infections

Supplemental Figure S2. Forrest plots on various fatty acids and COPD related outcomes

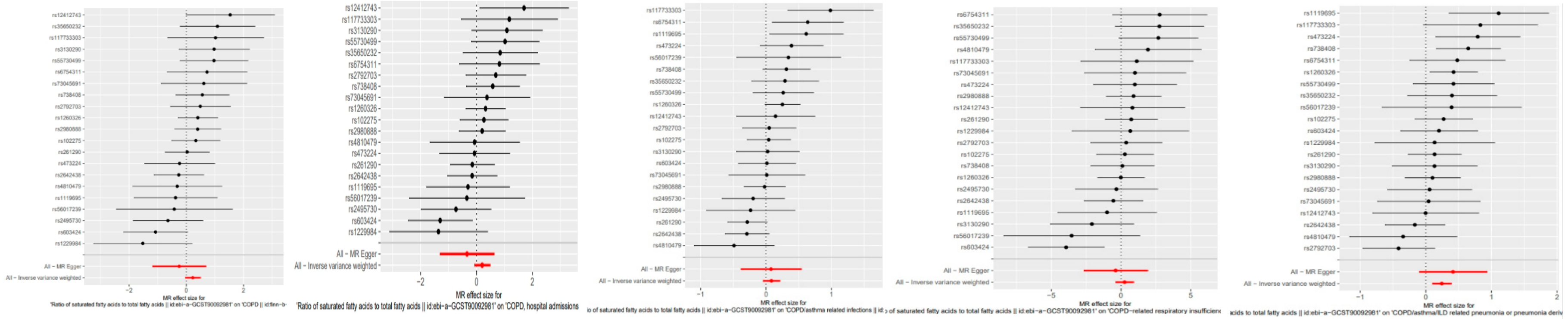

a) SFA/TFA and COPD, COPD hospitalization, COPD/asthma related infections, COPD insufficiency and pulmonary infections

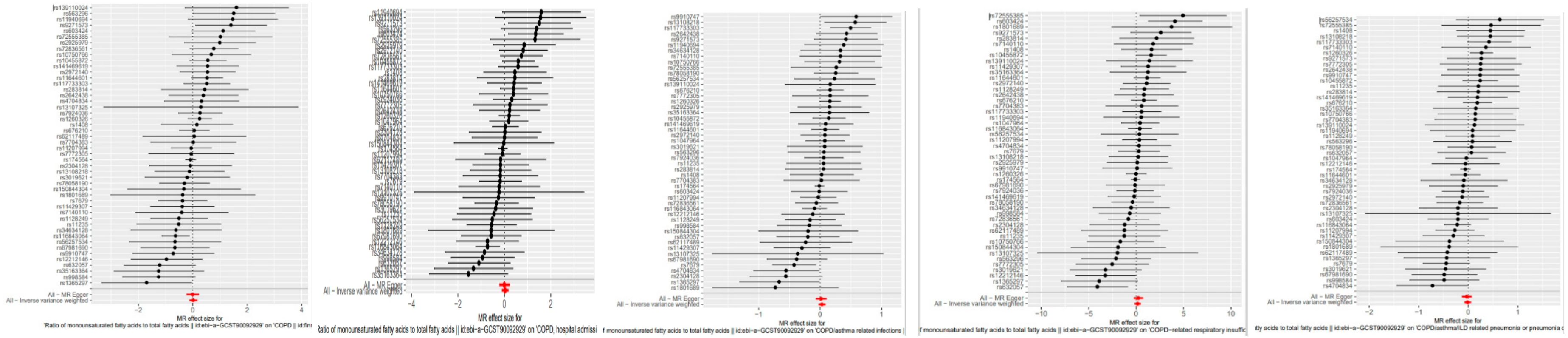

b) MUFA/TFA and COPD, COPD hospitalization, COPD/asthma related infections, COPD insufficiency and pulmonary infections

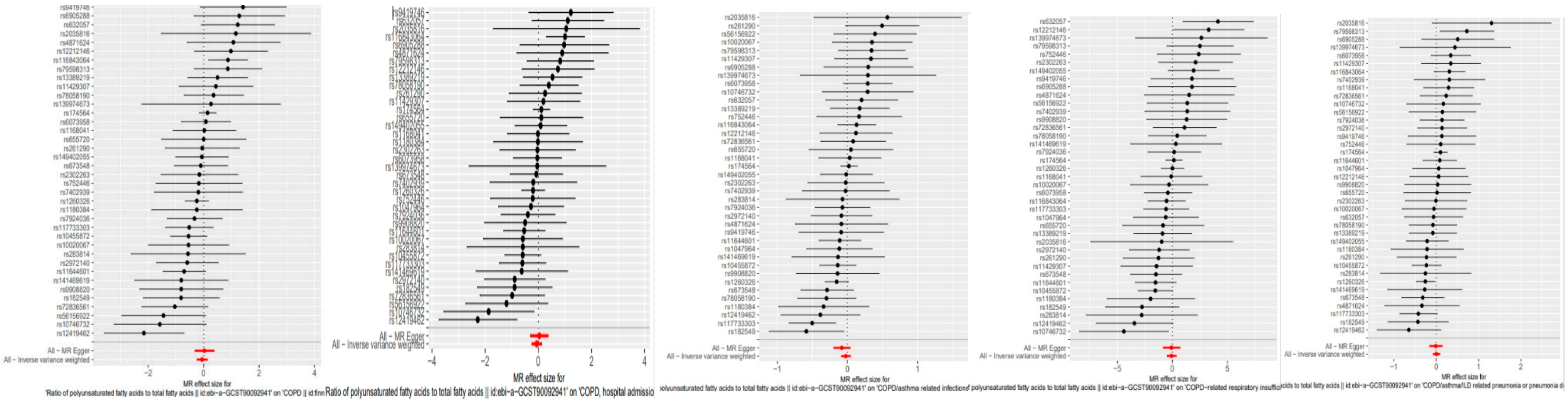

c) PUFA/TFA and COPD, COPD hospitalization, COPD/asthma related infections, COPD insufficiency and pulmonary infections

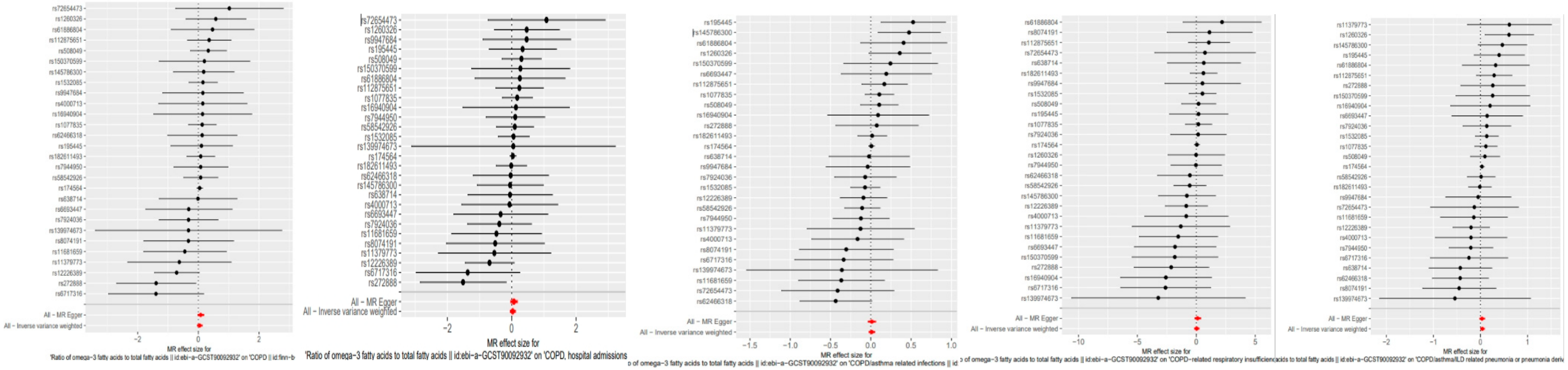

d) Omega-3/TFA and COPD, COPD hospitalization, COPD/asthma related infections, COPD insufficiency and pulmonary infections

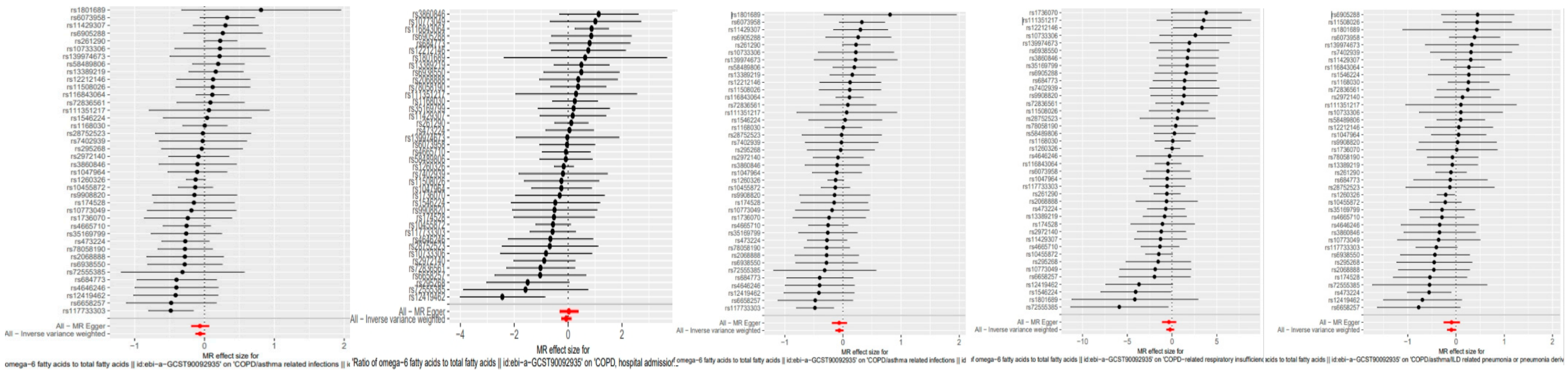

e) Omega-6/TFA and COPD, COPD hospitalization, COPD/asthma related infections, COPD insufficiency and pulmonary infections

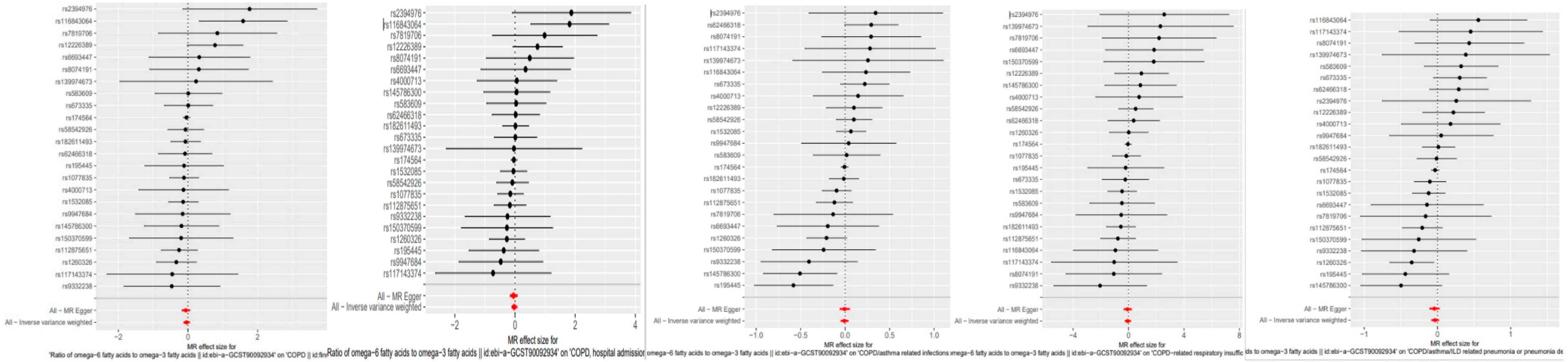

f) Omega-6/omega-3 and COPD, COPD hospitalization, COPD/asthma related infections, COPD insufficiency and pulmonary infections

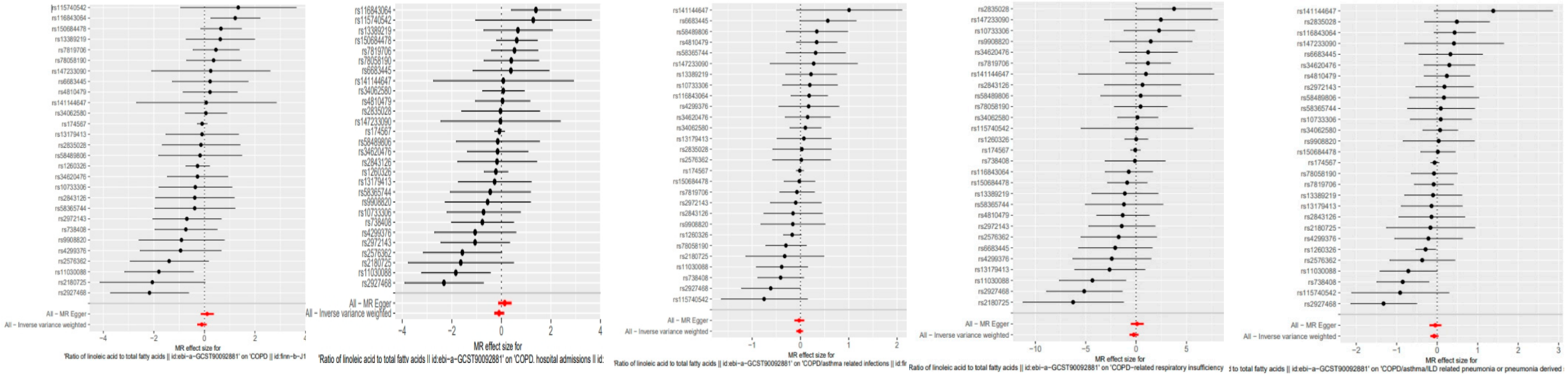

g) LA and COPD, COPD hospitalization, COPD/asthma related infections, COPD insufficiency and pulmonary infections

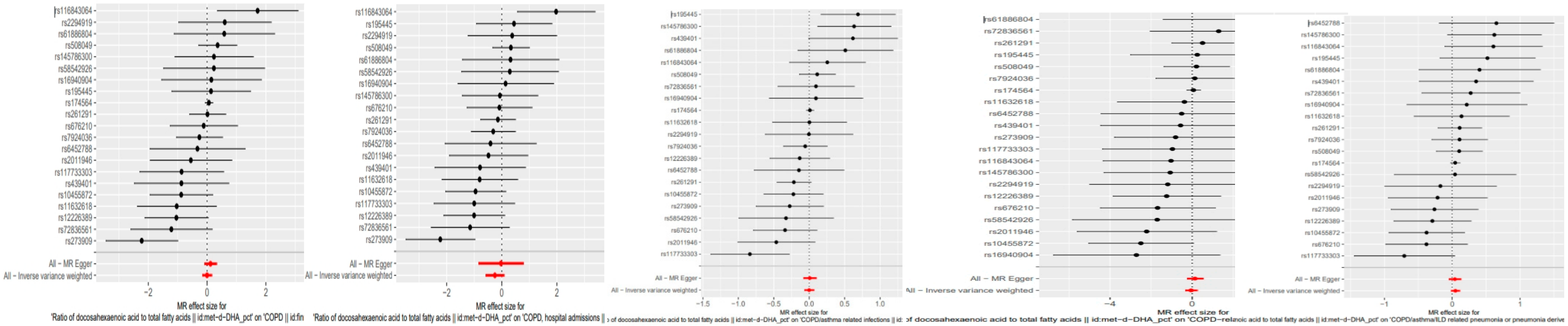

h) DHA and COPD, COPD hospitalization, COPD/asthma related infections, COPD insufficiency and pulmonary infections

Supplemental Figure S3. Leave-one-out plots on various fatty acids and COPD related outcomes

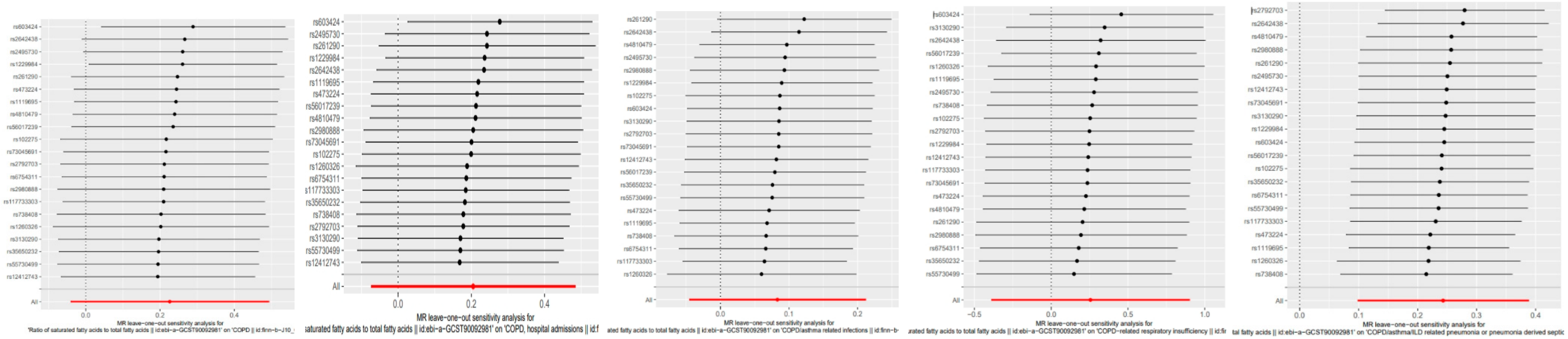

a) SFA and COPD, COPD hospitalization, COPD/asthma related infections, COPD insufficiency and pulmonary infections

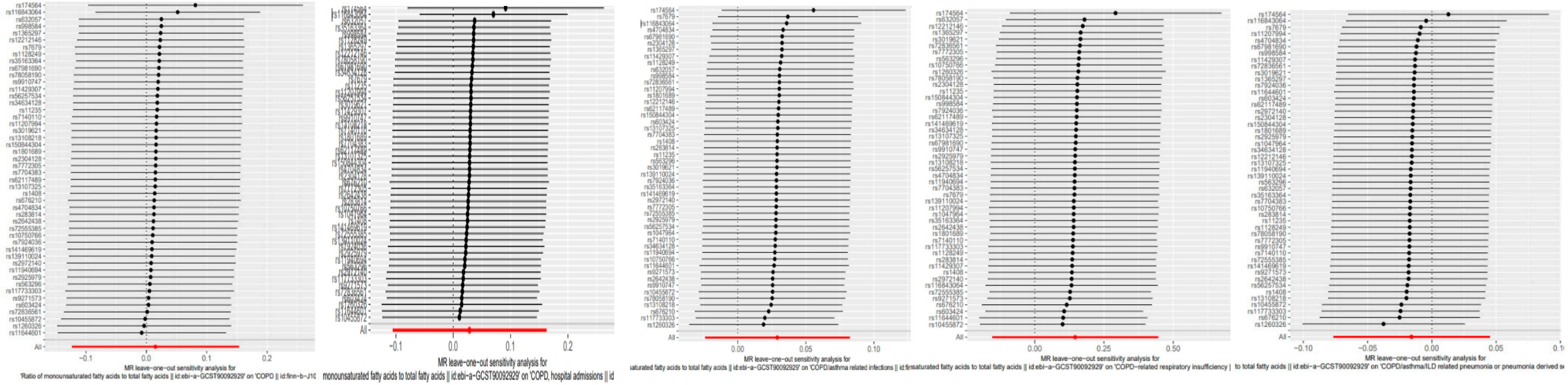

b) MUFA and COPD, COPD hospitalization, COPD/asthma related infections, COPD insufficiency and pulmonary infections

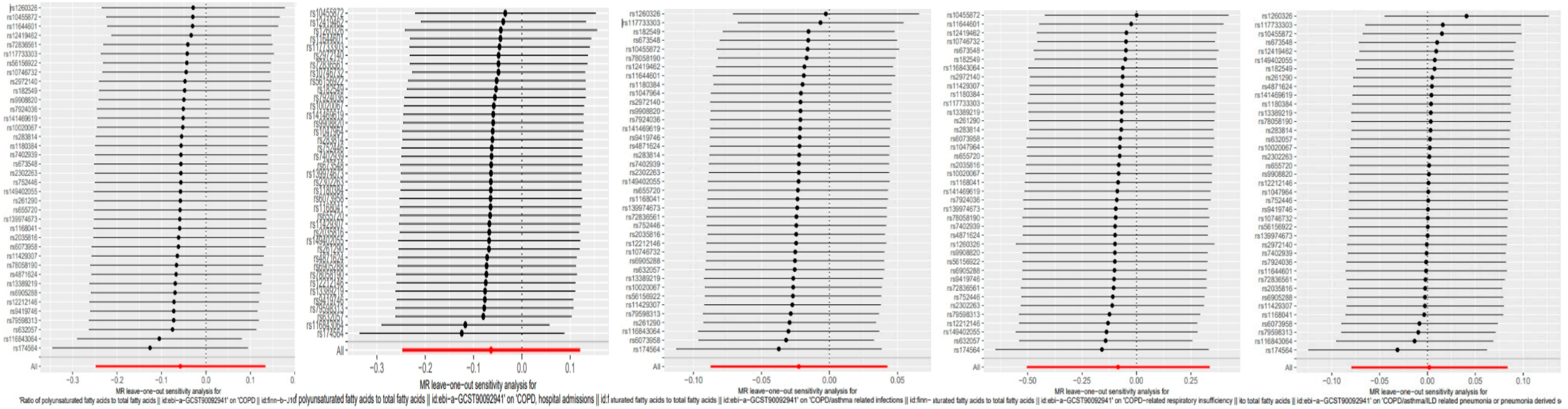

c) PUFA and COPD, COPD hospitalization, COPD/asthma related infections, COPD insufficiency and pulmonary infections

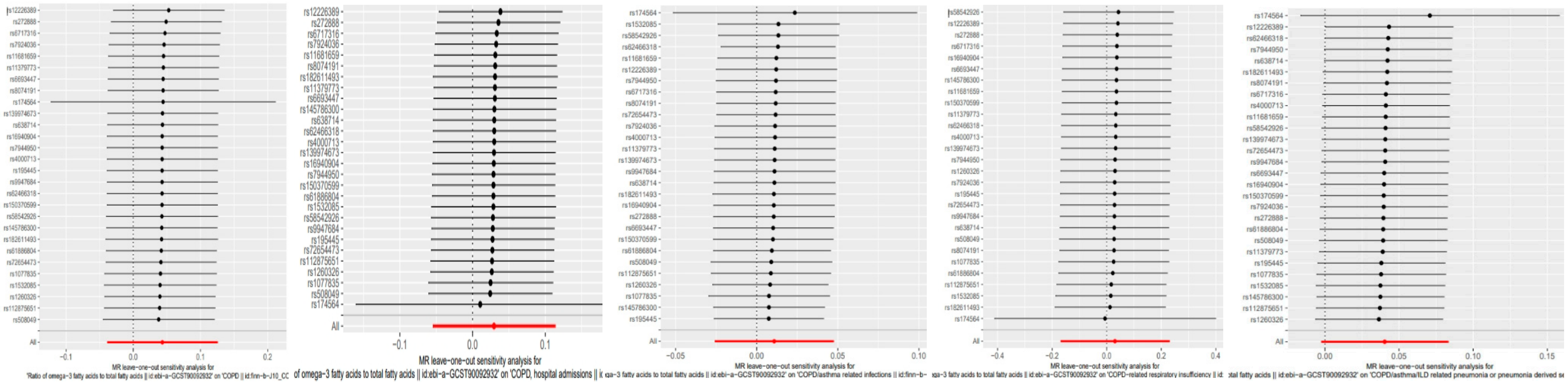

d) Omega-3 and COPD, COPD hospitalization, COPD/asthma related infections, COPD insufficiency and pulmonary infections

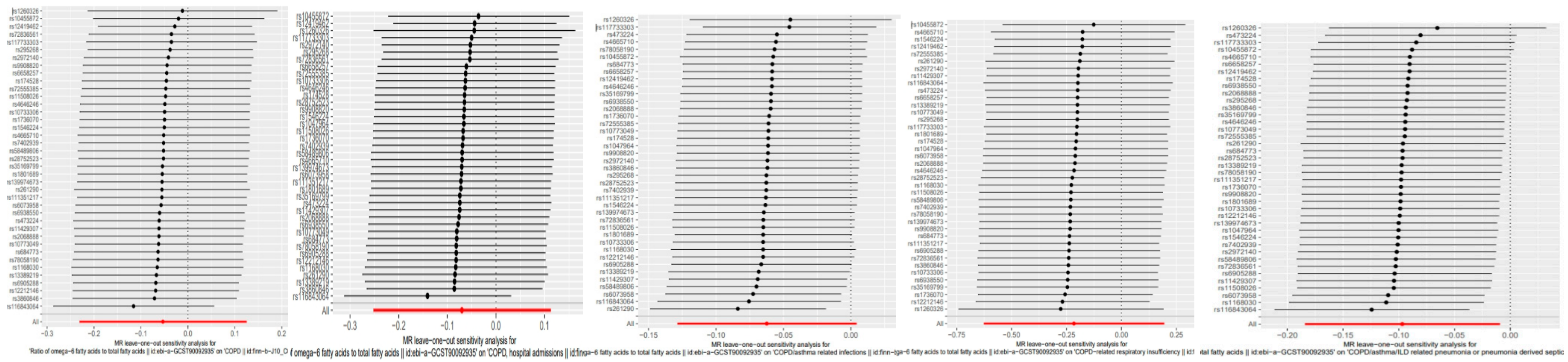

e) Omega-6 and COPD, COPD hospitalization, COPD/asthma related infections, COPD insufficiency and pulmonary infections

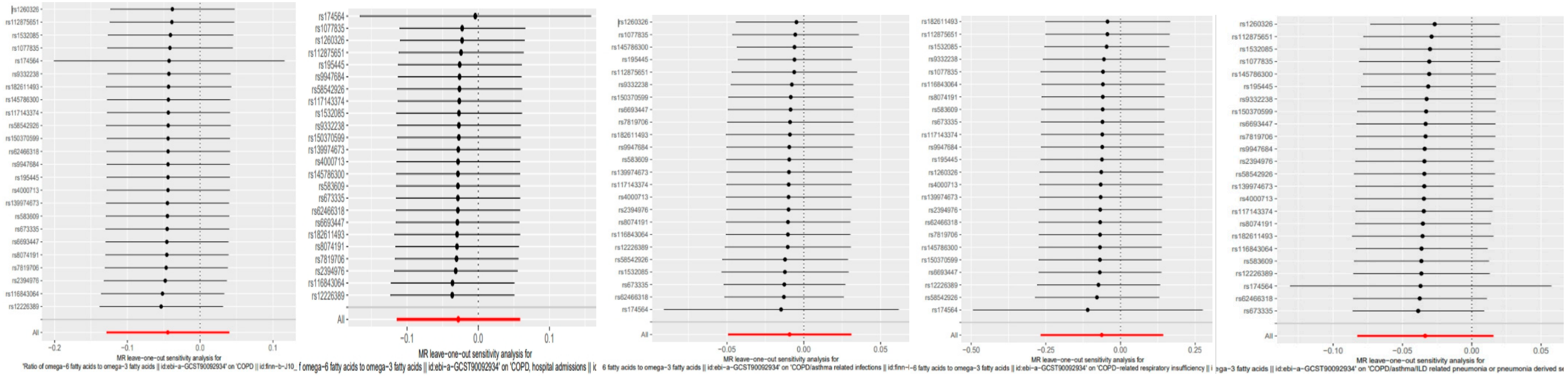

f) Omega6/omega-3 and COPD, COPD hospitalization, COPD/asthma related infections, COPD insufficiency and pulmonary infections

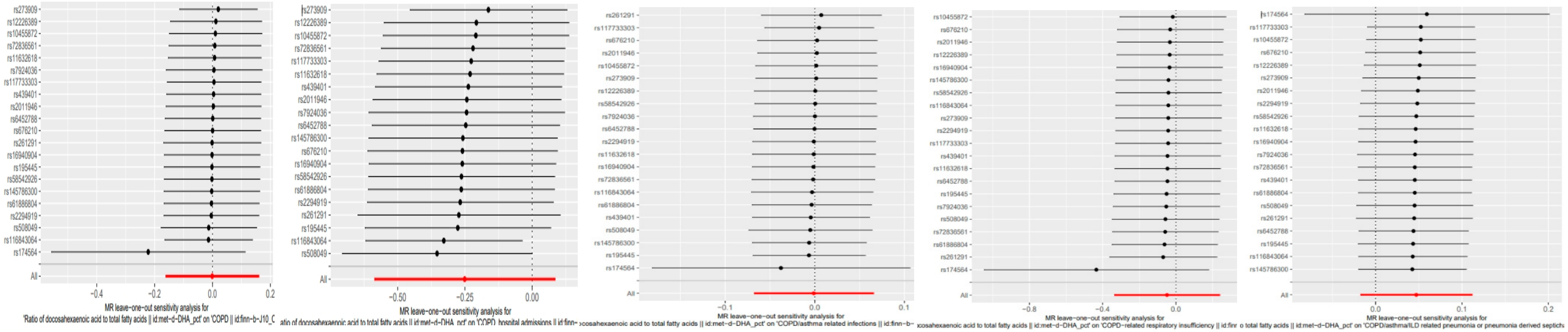

g) DHA and COPD, COPD hospitalization, COPD/asthma related infections, COPD insufficiency and pulmonary infections

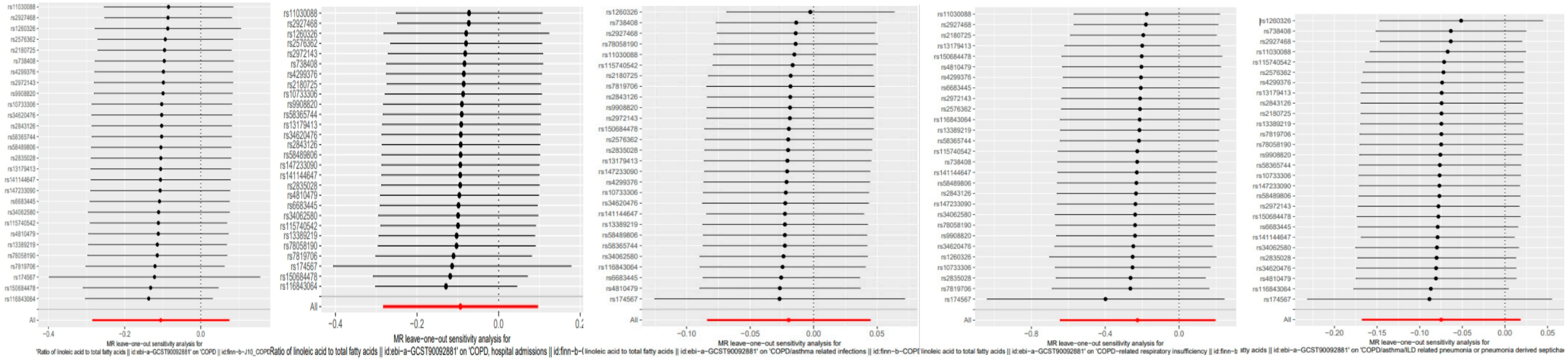

h) LA and COPD, COPD hospitalization, COPD/asthma related infections, COPD insufficiency and pulmonary infections

Supplemental Figure S4. Funnel plots on various fatty acids and COPD related outcomes

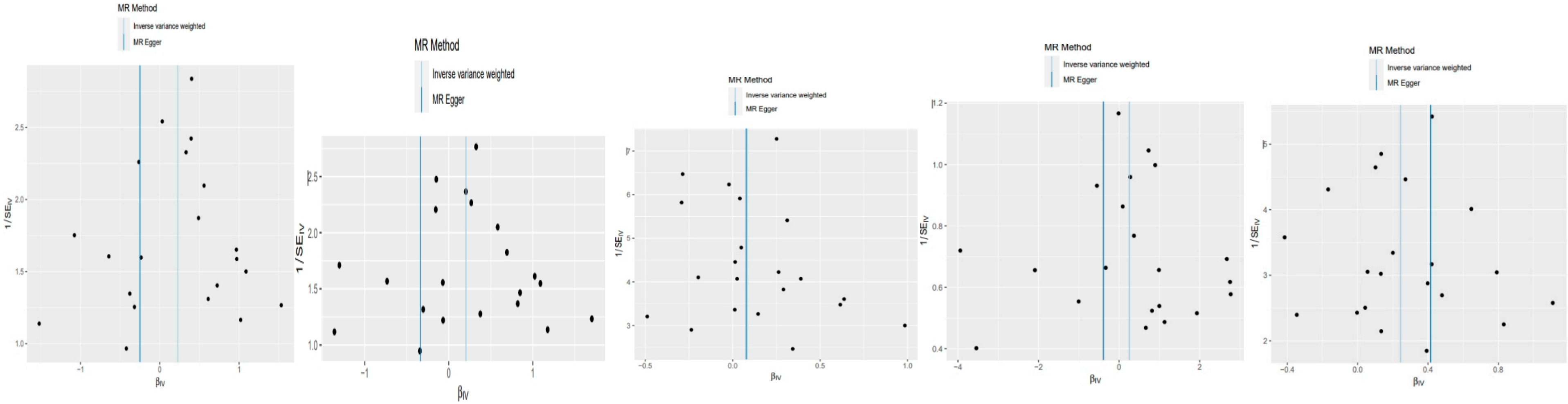

a) SFA and COPD, COPD hospitalization, COPD/asthma related infections, COPD insufficiency and pulmonary infections

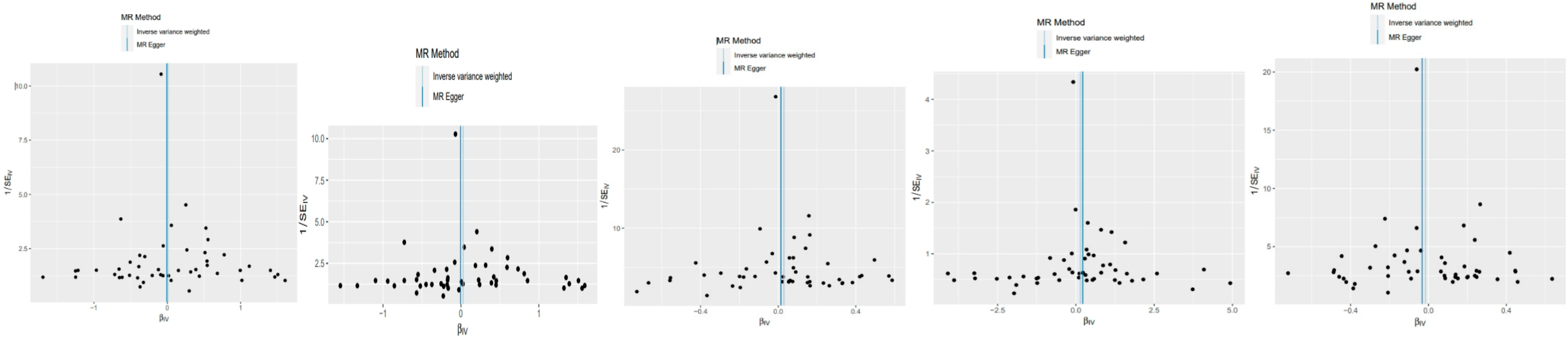

b) MUFA and COPD, COPD hospitalization, COPD/asthma related infections, COPD insufficiency and pulmonary infections

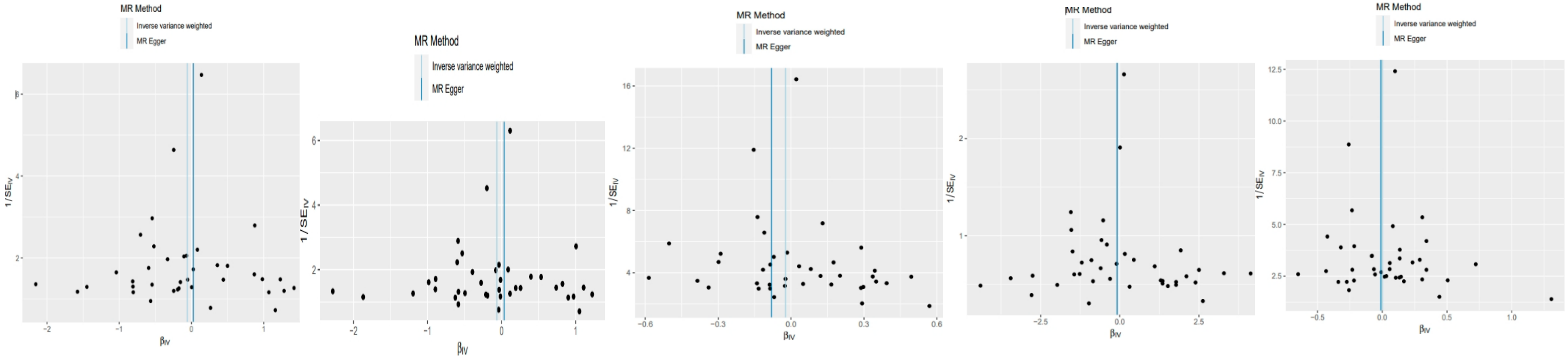

c) PUFA and COPD, COPD hospitalization, COPD/asthma related infections, COPD insufficiency and pulmonary infections

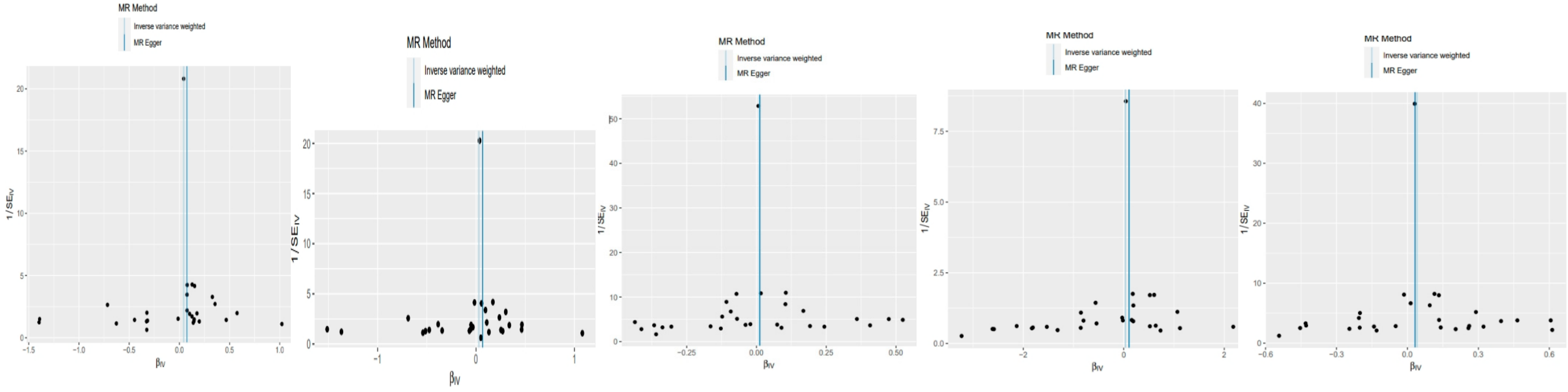

d) Omega-3 and COPD, COPD hospitalization, COPD/asthma related infections, COPD insufficiency and pulmonary infections

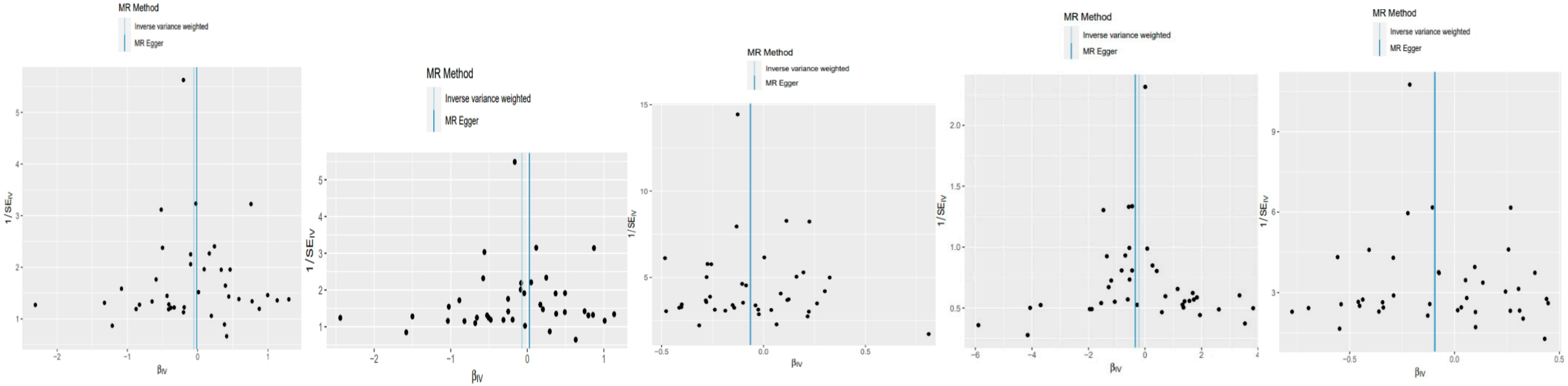

e) Omega-6 and COPD, COPD hospitalization, COPD/asthma related infections, COPD insufficiency and pulmonary infections

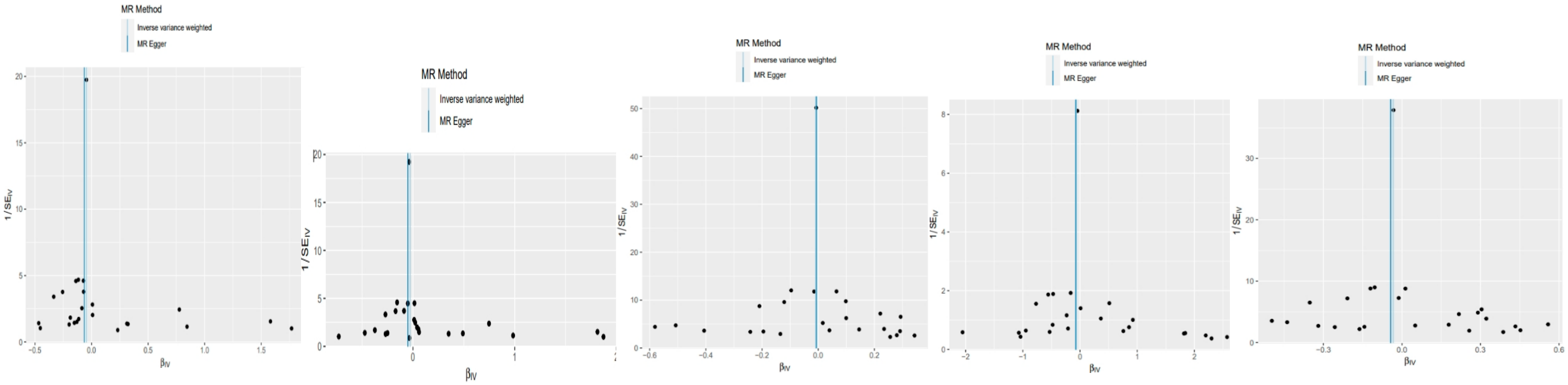

f) Omega-6/omega-3 and COPD, COPD hospitalization, COPD/asthma related infections, COPD insufficiency and pulmonary infections

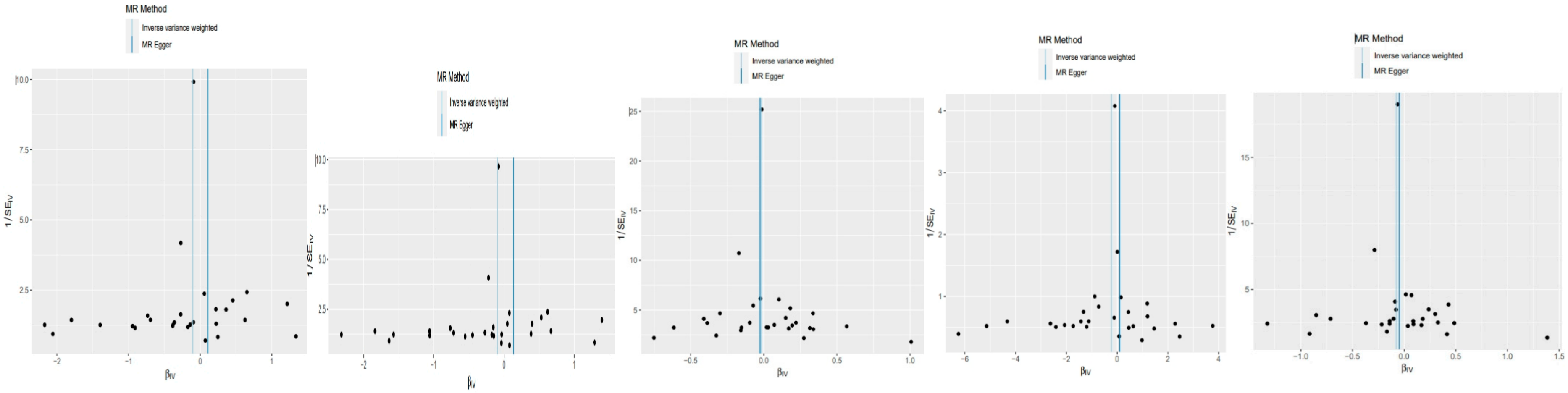

g) LA and COPD, COPD hospitalization, COPD/asthma related infections, COPD insufficiency and pulmonary infections

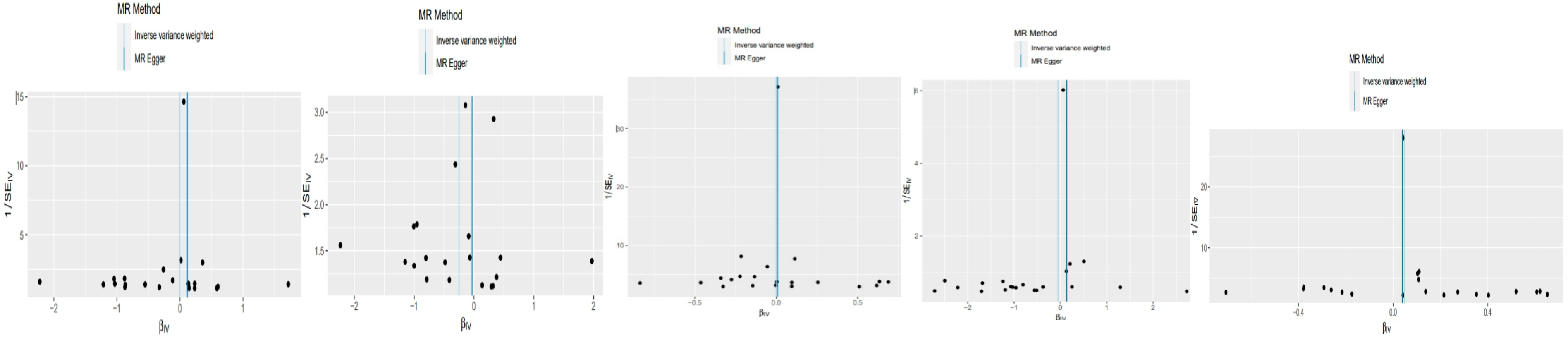

h) DHA and COPD, COPD hospitalization, COPD/asthma related infections, COPD insufficiency and pulmonary infections
